# Electrochemical oxidation of As(iii) on Pd immobilized Pt surface: kinetics and sensing performance†

**DOI:** 10.1039/c7ra12576c

**Published:** 2018-02-20

**Authors:** Md. Mahbubul Alam, Md. A. Rashed, Md. Musfiqur Rahman, Mohammed M. Rahman, Yuki Nagao, Mohammad A. Hasnat

**Affiliations:** Department of Chemistry, Shahjalal University of Science and Technology Sylhet–3114 Bangladesh mah-che@sust.edu mahtazim@hotmail.com +88-0821-715752 ext. 694 +88-0821-715752 ext. 694; Department of Chemistry, Mawlana Bhashani Science and Technology University Santosh, Tangail-1902 Bangladesh; School of Materials Science, Japan Advanced Institute of Science and Technology 1-1 Asahidai Nomi Ishikawa 923-1292 Japan; Centre of Excellence for Advanced Materials Research (CEAMR), Chemistry Department, Faculty of Science, King Abdul Aziz University P.O. Box 80203 Jeddah 21589 Saudi Arabia

## Abstract

Pd nanoparticles were electrochemically immobilized on a Pt surface in the presence of sodium dodecyl sulfate (SDS) molecules to study the electrokinetics of arsenite oxidation reactions and the corresponding sensing activities. The X-ray photoelectron spectroscopy (XPS) analysis showed that on the Pt surface, Pd atoms exist as adatoms and the contents of Pd(0) and Pd(ii) were 75.72 and 24.28 at%, respectively, and the particle sizes were in the range of 61–145 nm. The experimental results revealed that the catalytic efficiency as well as the charge transfer resistance (at the redox potential of the Fe(ii)/Fe(iii) couple) increased in the order of Pt < Pt–Pd < Pt–Pd_sds_. A Pt–Pd_sds_ electrode exhibited an open circuit potential (OCP) of 0.65 V in acidic conditions; however, when 50.0 mM NaAsO_2_ was present, the OCP value shifted to 0.42 V. It has been projected that the As(iii) oxidation proceeds using a sequential pathway: As(iii) → As(iv) → As(v). After optimization of the square wave voltammetric data, the limits of detection of As(iii) were obtained as 1.3 μg L^−1^ and 0.2 μg L^−1^ when the surface modification of the Pt surface was executed with Pd particles in the absence and presence of the SDS surfactant, respectively. Finally, real samples were analyzed with excellent recovery performance.

## Abstract 2

Amplification of true surface area can be improved when Pd particles are deposited on a substrate in the presence of sodium dodecyl sulfate (SDS) surfactant. In acidic medium, As(iii) undergoes a two-step oxidation process.

## Graphical abstract

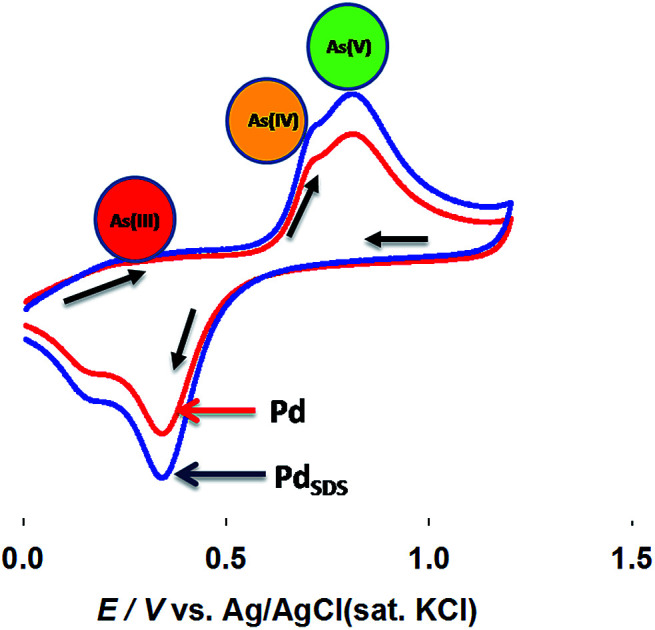

## Introduction

Arsenic is a highly toxic element and its compounds are known to be poisonous substances, which are widely distributed in the earth's crust. There are up to 50 million people in the world at the risk of arsenicosis, because of which the World Health Organization (WHO) addressed this problem as the world's worst mass poisoning disaster.^1–3^ Prolonged ingestion of arsenic contaminated water can have a variety of adverse effects on health, including skin, lung, heart, bladder cancer and kidney diseases.^3,4^ In many countries, including Argentina, Bangladesh, Chile, China, India (West Bengal), Mexico, Thailand, and the United States of America, natural contamination of arsenic is a serious health concern. Hence, the WHO and the US Environmental Protection Agency (EPA) have revised the maximum value of arsenic concentration in drinking water from 50 μg L^−1^ to 10 μg L^−1^.^5,6^ The toxicity of arsenic compounds depends on their forms. Inorganic compounds are about 100 times more toxic than organic metabolites. Inorganic arsenic exists in water primarily in two forms: arsenite (AsO_2_^−^) and arsenate (AsO_4_^3−^). The arsenite ions are formed under strongly reduced conditions and are extremely soluble in water and approximately 60 times more toxic than the arsenate ions due to their ability to react with enzymes in the human respiratory system.^7^ The high toxicity and widespread occurrence of arsenic in ground water have prompted a pressing need for developing methods and materials for arsenic monitoring. A wide variety of methods, such as atomic absorption spectrophotometry (AAS), X-ray spectrometry, electrothermal AAS in graphite furnaces (ETAAS), inductively coupled plasma-mass spectroscopy (ICP-MS), capillary electrophoresis, neutron activation analysis (NAA), UV-Visible spectroscopic, ion exchange, and colorimetry, have been used to determine arsenic in water.^8–13^ In contrast, the versatile electrochemical technique offers some advantages including simple instrumentation and operation, low cost and rapid analysis, high sensitivity and minimum and/or no secondary pollution. A large number of studies have been carried out on arsenic detection and low limit of determination (LOD) using electrooxidative^14–17^ and stripping voltammetric methods.^18–21^ Its noteworthy that the principle of the oxidation process is based on the conversion of As(iii) into a stable product As(v) by releasing two electrons.^22–24^ However, since As(iii) species are more soluble and mobile than As(v) species, development of electrode materials for the conversion of As(iii) to As(v) regarding detection, determination, and removal is still a challenge. To date, several articles have been published on the electrochemical and electrokinetic studies of As(iii) electro-oxidation using Pt, Au, Hg, indium tin oxide (ITO) coated glass and carbon-based materials.^16,17,22–31^ In this context, to the best of our knowledge, very few research groups have worked on the applicability of palladium as an electrocatalyst for As(iii) electro-oxidation.^22^ Moreover, contemporary scientists and technologists are paying much attention to minimizing the usage of precious metals as catalysts by developing porosity in the catalytic matrix since this approach can improve the active catalytic area. In this concern, Attard *et al.*, reported that mesoporous Pt films with a large surface area could be fabricated onto Au surface if the deposition is executed using hexachloroplatinic acid (H_2_[PtCl_6_]) in the presence of an octaethylene glycol monohexadecyl ether (C_16_EO_8_) surfactant.^32^ Another surfactant, sodium dodecyl sulphate (SDS), has also been proved to be an effective templating reagent in generating larger surface area pertaining to the development of large capacity oxygen storage mesoporous materials^33^ and in attaining effective nitrite reduction when the Pt electrode is fabricated on a glassy carbon substrate.^34^ Since the Pt surface is highly corrosion resistant and one of the most extensively used electrode substrates, we amplified the active surface area by immobilizing Pd particles on it using a very simple electrochemical approach. In the present study, we immobilized Pd nanoparticles on a platinum substrate in the presence of an SDS surfactant assuming that this approach might improve the sensing activity of the electrode surface in the context of arsenite oxidation reactions.

Herein, prior to applying the Pd fabricated Pt electrode as a sensor to detect As(iii), the catalytic surface was characterized using different analytical tools. Field emission scanning electron microscopy (FE-SEM), X-ray photoelectron spectroscopy (XPS) and electrochemical (electrochemical impedance spectroscopy (EIS) and cyclic voltammetry (CV)) techniques were used. In addition, the heterogeneous kinetics of the arsenite electro-oxidation reactions was also studied in 0.1 M H_2_SO_4_ by applying convolution potential sweep voltammetry (CPSV) for understanding the arsenite oxidation mechanism.

## Experimental

### Materials and chemicals

All the chemicals were of analytical grade and were used without further purification. Palladium nitrate (Pd(NO_3_)_2_) was obtained from Wako Pure Chemical Industries Ltd., Japan. Sulfuric acid (H_2_SO_4_), alumina powder (Al_2_O_3_), and potassium ferrocyanide (K_4_[Fe(CN)_6_]) were purchased from Aldrich Chemical Co. Inc., Germany and sodium meta arsenite was purchased from Merck, Germany. All the solutions were prepared with Millipore Milli-Q water (resistivity >18 MΩ cm and micro-organic concentration ≤3 μg L^−1^). Degassing was conducted prior to each measurement using pure N_2_.

### Preparation of Pt–Pd catalysts

Before conducting the experiments, the bare Pt electrode was mechanically polished with alumina (0.3 μm) slurry on a soft lapping pad until a mirror-like shiny surface was obtained. Then, the polished surface was rinsed with double-distilled water and cleaned consecutively using ethanol and double-distilled water with sonication (10 min each). Finally, the Pt surface cleaning was carried out by potential cycling in a N_2_-saturated 0.5 M H_2_SO_4_ solution from −0.2 to 1.5 V at a scan rate of 100 mV s^−1^ until the characteristic reproducible voltammograms of cleaned Pt surface pertaining to hydrogen adsorption/desorption were obtained.

The Pd particles were then electrochemically deposited on the Pt surface by means of cyclic voltammetry. For this, potential cycling was performed using a Pt disk electrode (2 mm diameter) between 0 and −1.0 V 10 times at a 100 mV s^−1^ scan rate in N_2_-saturated 0.01 M Pd(NO_3_)_2_ in the absence and the presence of 100 mg L^−1^ SDS. A well-defined cathodic peak was observed at −0.85 V corresponding to the electro-deposited Pd metals onto the Pt substrate according to the eqn (1).

1 Pd^2+^ + 2e^−^ → Pd

After successful deposition of Pd on the Pt surface, the electrode was cleaned thoroughly with copious amounts of deionized water to remove loosely bonded particles (including SDS) and then dried at 60 °C prior to electrochemical investigations. In this article, the Pd modified Pt electrodes fabricated in the absence and in the presence of SDS molecules were designated as Pt–Pd and Pt–Pd_sds_, respectively.

### Surface characterization

The morphology of the Pd modified surface was evaluated using a field emission scanning electron microscopic (FE-SEM) instrument (JSM-7600F, Japan). Energy dispersive X-ray analysis (XEDS) was performed using a FESEM-coupled XEDS from JEOL, Japan. The XPS study was performed using a DLD spectrometer (Kratos Axis-Ultra; Kratos Analytical Ltd.) with an Al Kα radiation source (1486.6 eV). Energy calibration and component separation were conducted using the bundled software with pure Gaussian profiles and a Shirley background.

### Electrochemical measurements

Electrochemical investigations were carried out with a computer-controlled Autolab potentiostat (PGSTST128 N, Netherlands) using a conventional three electrode cell. A Pd-modified Teflon jacket-coated Pt disk electrode (2 mm in diameter) served as the working electrode with Ag/AgCl (3 M KCl) and Pt wire acting as reference and counter electrodes, respectively. All the experiments were carried out at room temperature (25 ± 0.5 °C) in 0.1 M H_2_SO_4_.

The working electrode (Pt/Pt–Pd/Pt–Pd_sds_) along with the counter and the reference electrodes were merged to form a 3-electrode cell containing 0.1 M H_2_SO_4_ with various concentration of As(iii). The effect of scan rate was investigated by varying the scan rates, while keeping the concentration of As(iii) (0.25 mM) constant. EIS measurements were performed in N_2_-saturated 0.1 M H_2_SO_4_ solution containing 1 mM NaAsO_2_ at an exciting potential of 1.0 V. The frequency range of EIS measurements was 0.1 MHz to 0.1 Hz with 50 measuring points. The open circuit potential (OCP) of the Pt–Pd_sds_ electrode was determined in 0.1 M H_2_SO_4_ solution in the absence and the presence of 50 mM As(iii) by recording linear polarization curves. The square wave voltammograms (SWV) were recorded (between 1 and 225 μM As(iii) concentration) under inert conditions by applying an optimized step potential of 8 mV, amplitude of 20 mV, and frequency of 25 Hz.

## Results and discussion

### Surface characterization

**Fig. 1:**
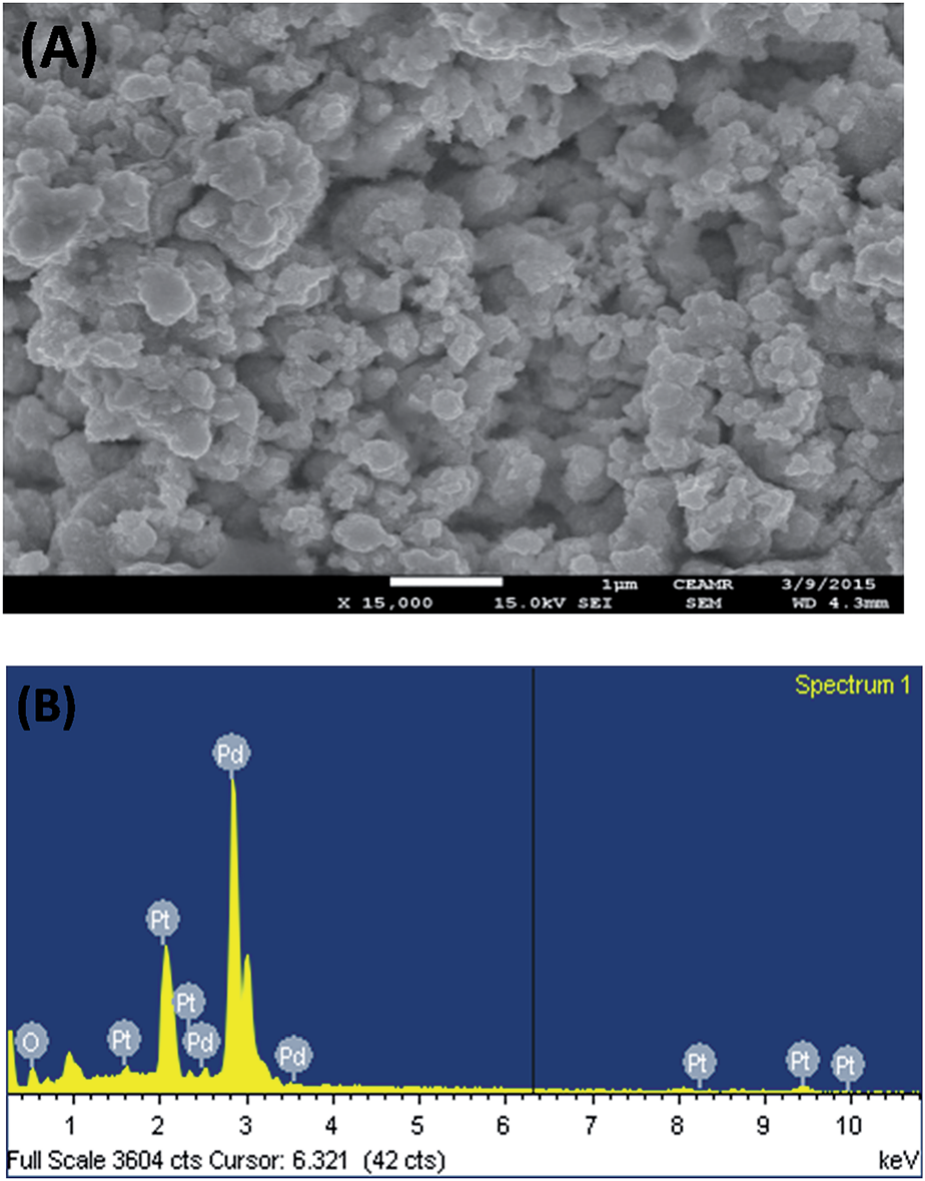
FE-SEM image (A) and EDS spectrum (B) of the Pd particles deposited on Pt surface.

**Fig. 2:**
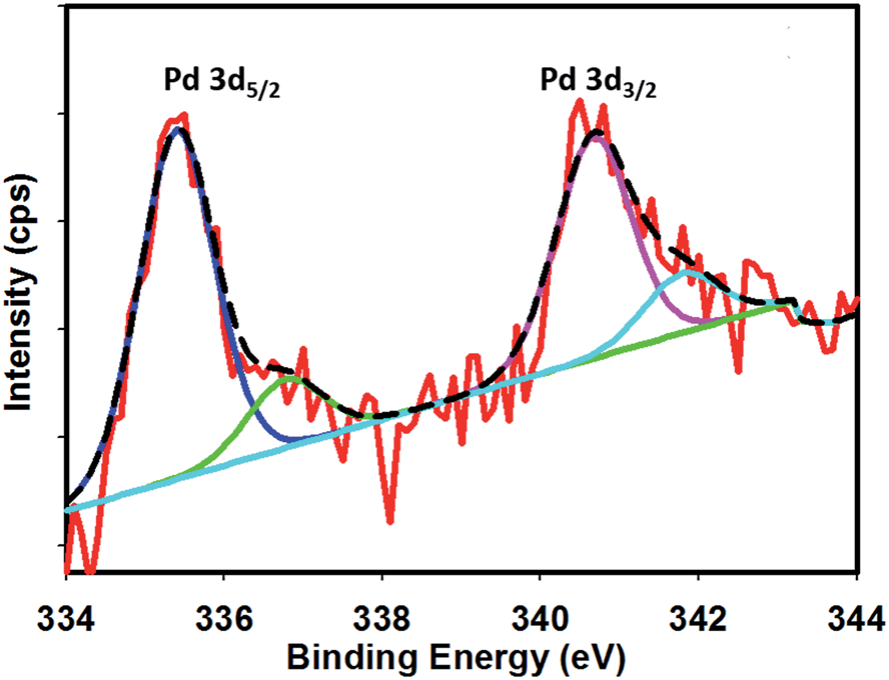
XPS fine spectra of Pd particle deposited on the Pt surface.

Fig. 1 shows the FE-SEM image with associated EDS spectra of Pd particles deposited on the Pt surface. It can be observed that the deposited particles are globular in nature and exist in aggregated form. The particle sizes of the deposited Pd particles are in the range of 61–145 nm. The intense EDS peak related to Pd particles as compared to that of Pt particles indicates that the surface predominantly contained Pd particles. In order to examine the surface properties profoundly, subsequent XPS analysis was performed. Fig. 2 shows the XPS fine scan spectra of Pd particles immobilized on the Pt surface. The XPS spectra (Fig. 2) of the deposited Pd particles over the Pt surface show two doublets originating from the spin–orbital splitting of the 3d_5/2_ and 3d_3/2_ states with the peaks centred at 335.4 eV and 340.7 eV, respectively, which are well-matched with the literature values.^35^ The peak corresponding to Pd 3d_5/2_ represents Pd(0) (BE = 335.4 eV) and an additional shoulder-like peak indicates the existence of Pd(ii) species (BE = 337.5 eV), which is higher than the reported value (336.9 eV) for Pd(ii).^36^ Thus, we assumed that this shifting of BE appeared due to the existence of some free Pd^2+^ ions adhered on the surface during the electrochemical deposition of palladium from Pd(NO_3_)_2_ solution. The narrow FWHM value (1.11 eV) suggests the identical oxide forms of Pd particles. The deconvolution of the spectrum suggested that the content of Pd(0) and Pd(ii) particles were 75.72 and 24.28 at%, respectively. Moreover, to examine any change in Pt properties due to Pd deposition, the XPS of the Pt particles was recorded before and after Pd deposition. Before Pd deposition, the Pt particles exhibited a doublet at 71.2 eV (4f_7/2_) and 74.5 eV (4f_5/2_), indicating metallic platinum (Pt(0)) (see the ESI, Fig. S1†). After deconvolution, the shoulder-like peaks shifted to higher binding energies, *i.e.*, 72.4 eV and 75.5 eV, which could be assigned to the existence of Pt(iv) species as platinum oxide and/or hydroxide that might have been formed due to oxidation of Pt(0) particles. After deposition of Pd particles, the position of the major peaks binding energies (associated to Pt 4f_7/2_ and Pt 4f_5/2_) were not measurably altered (see the ESI, Fig. S2†), indicating that Pt atoms did not have any chemical interactions with the deposited Pd atoms.

Thus, we assumed that Pd atoms existed on the Pt surface as adatoms. To detect the Pt(iv) species, we tried to deconvolute the spectrum. However, the peak intensity of the Pt atoms was significantly low and spectra deconvolution was not possible. This observation suggests that the Pt surface was buried sufficiently under the Pd layers. Thus in this article, we have designated the working electrode as the Pd modified Pt electrode.

### Electrochemical characterization

**Fig. 3:**
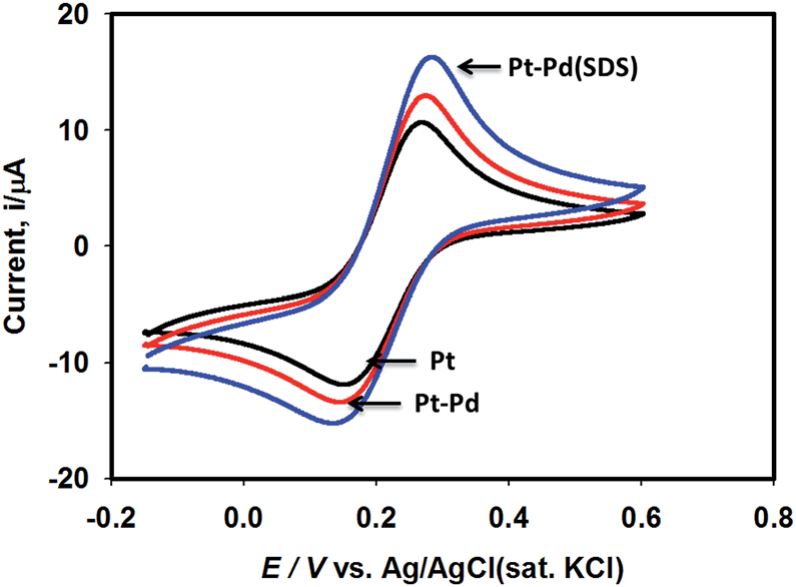
Cyclic voltammograms of 0.01 M K_4_Fe(CN)_6_ in 0.1 M KNO_3_ using Pt, Pt–Pd and Pt–Pd_sds_ electrodes recorded at scan rate of 50 mV s^−1^.

**Fig. 4:**
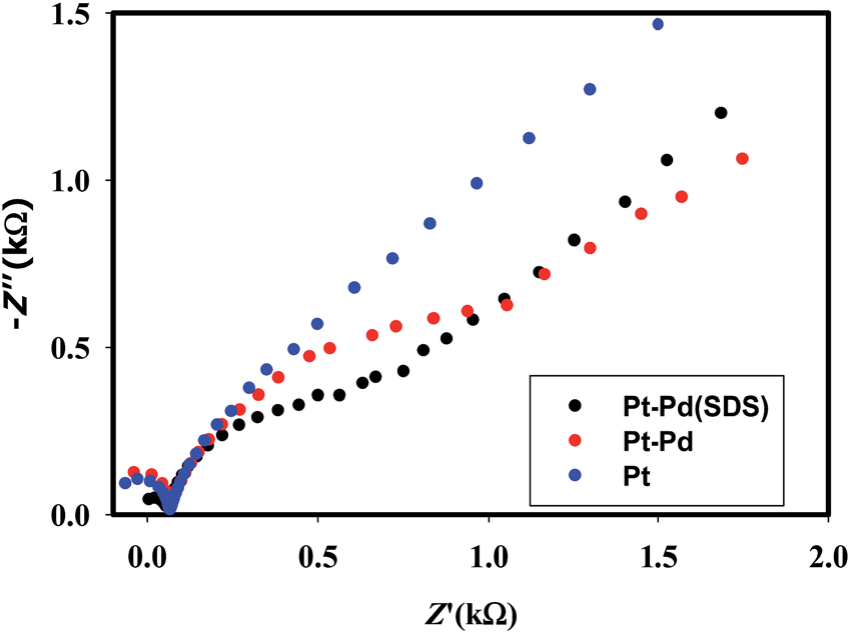
EIS spectra of 1 mM NaAsO_2_ in 0.1 M H_2_SO_4_ using Pt, Pt–Pd and Pt–Pd_sds_ electrodes recorded at an excitation potential of 1.0 V.

Fig. 3 shows the voltammograms of 0.01 M K_4_Fe(CN)_6_ in 0.1 M KNO_3_ recorded using Pt, Pt–Pd, and Pt–Pd_sds_ electrodes at a scan rate of 50 mV s^−1^. The Pd particles were deposited on a Pt surface by cycling a Pt disk electrode in 0.01 M Pd(NO_3_)_2_ at the scan rate of 100 mV s^−1^ between −0.4 and −1.2 V (ESI, Fig. S3†). On integrating the peak area, it was found that the content of the deposited Pd particles was 2.7 × 10^─3^ μg, which was not affected by the presence or absence of the SDS molecules. Nevertheless, the Pt–Pd_sds_ electrode generated maximum redox currents and followed a trend of Pt < Pt–Pd < Pt–Pd_sds_ under the same experimental condition. It is well known that the efficiency of a redox process on a catalytic surface is generally increased by increasing the active surface area.^37–40^ Due to smaller sizes, the Pd nanoparticles provided higher surface area and thus improved contact areas with the reactants; this observation is analogous to those in homogeneous catalytic processes. However, it is observed that if the Pd deposition is executed in the presence of SDS molecules, the peak currents, in reference to a Pt electrode, increased by *ca.* 48%, while it increased by only 27% if the electrode was fabricated in the absence of SDS molecules. This observation implies that the Pd nanoparticles deposited in the presence of SDS molecules provided more contact area to the reactants than the Pd nanoparticles deposited in the absence of SDS molecules. Thus, in conjunction with reference,^32^ it can be assumed that the porosity of the catalytic matrix probably developed when the Pd nanoparticles were deposited in the presence of SDS molecules, thus providing more active surface area. In order to explore the consequences of surface area enlargement, the impedance spectra of the 1 mM NaAsO_2_ in 0.1 M H_2_SO_4_ was recorded by applying 1.0 V as the excitation potential assuming that at this potential, As(iii) is oxidized. Fig. 4 shows the classical presentation of the Nyquist plots recorded using Pt, Pt–Pd, and Pt–Pd_sds_ electrodes. As shown in Fig. 4 the charge transfer resistance (*R*_ct_) is augmented with an increasing order of Pt–Pd_sds_ < Pt–Pd < Pt. The least *R*_ct_ value exhibited by Pt–Pd_sds_ confirms that the most favourable charge transfer pertaining to As(iii) oxidation is administered by this electrode.

**Fig. 5:**
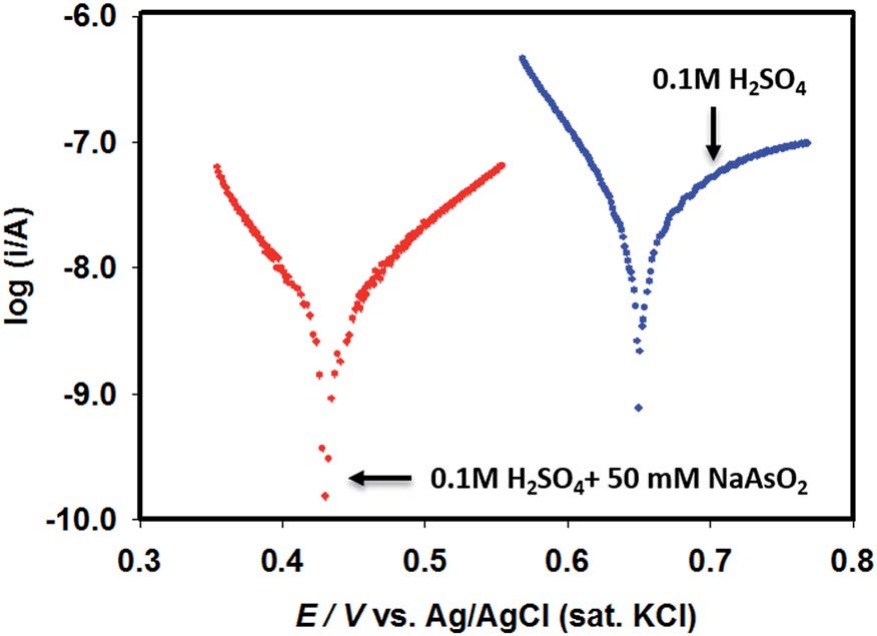
Linear polarization curves of Pt–Pd_sds_ electrode recorded in 0.1 M H_2_SO_4_ in absence and in presence of 50 mM NaAsO_2_.

Since Pt–Pd_sds_ assembly appeared to be the most efficient catalyst, in order to evaluate the types of interactions prevailing between Pd particles and the target arsenite ions in open circuit conditions in 0.1 M H_2_SO_4_, linear polarization curves were recorded using a Pt–Pd_sds_ electrode assembly as a working electrode as shown in Fig. 5. It can be observed that the Pt–Pd_sds_ surface exhibited OCP at 0.65 V; however, when 50 mM NaAsO_2_ was present, the OCP value shifted to 0.42 V. This observation suggests that Pt–Pd_sds_ surface received negative charges after adsorption of arsenite ions. Thus, it could be assumed that Pt–Pd_sds_ electrode might catalyse arsenite oxidation reactions after a pre-adsorption step. Therefore, to investigate the reaction mechanism in detail, heterogeneous electron transfer kinetics was studied next.

### Arsenite oxidation kinetics

**Fig. 6:**
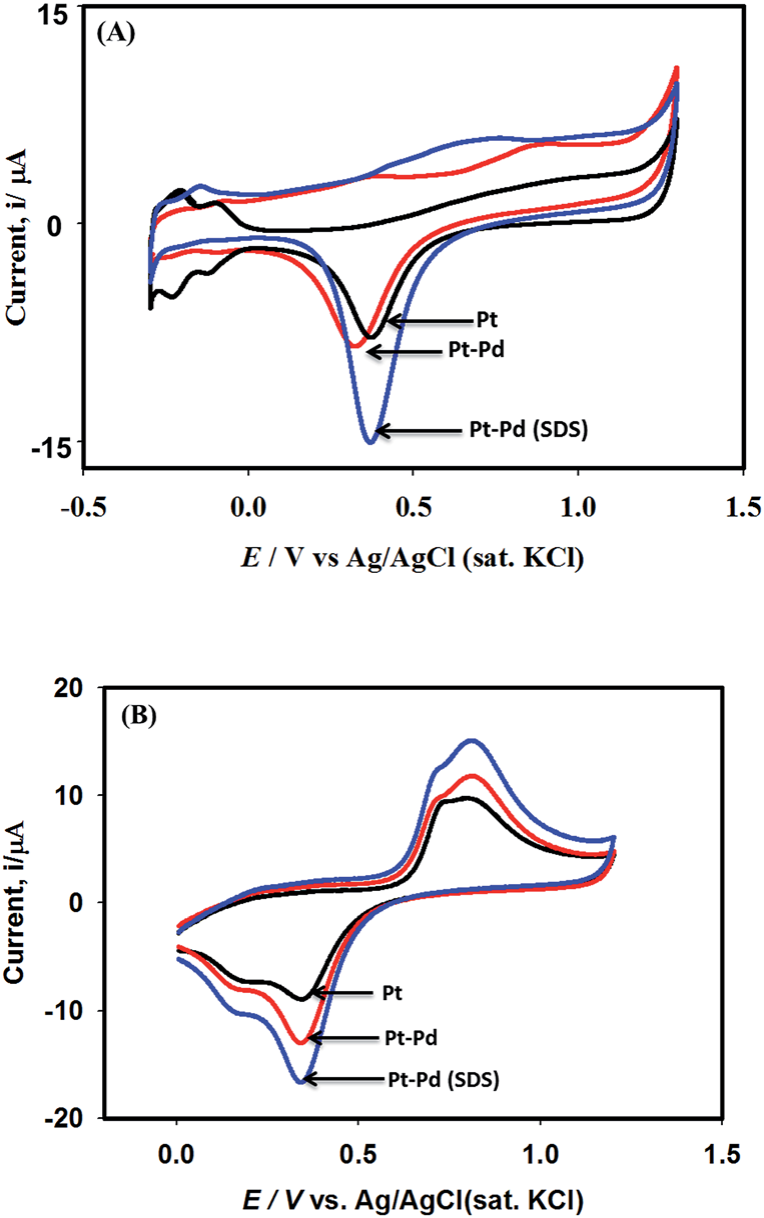
Cyclic voltammograms of 0.1 M H_2_SO_4_ using Pt, Pt–Pd and Pt–Pd_sds_ electrodes at a scan rate of 100 mV s^−1^ (panel A). Panel ‘B’ shows corresponding voltammograms in presence of 0.25 mM NaAsO_2_.

Fig. 6 shows the cyclic voltammograms of 0.1 M H_2_SO_4_ in the absence and in the presence of 0.25 mM NaAsO_2_ recorded using Pt, Pt–Pd and Pt–Pd_sds_ electrodes at a scan rate of 100 mV s^−1^. The electro-oxidation reactions of arsenite ions were studied by scanning the test solution between 0 and 1.2 V. A control experiment was performed and the result is included in the ESI (Fig. S4†).

As shown in Fig. 6A, during the forward scan (negative to positive direction) small amount of oxidation current is observed between 0.5 and 1.1 V because of oxide formation; above 1.1 V, the current displayed is clearly associated with water oxidation. However, when the scan is reversed from 1.2 V, well-defined reductive waves (centred at *ca.* 0.34 V) are observed, which could be attributed to the reduction of the oxide particles formed during the positive scan. It should be noted that the peak height as well as the peak area obtained by the Pt–Pd_sds_ electrode are larger than those obtained by a Pt or Pt–Pd surface, which again ensured enlargement of the active area of the Pd surface when deposition was executed in the presence of the SDS surfactant. However, when arsenite ions were added to 0.1 M H_2_SO_4_ solution, during positive scan, the characteristic waves pertaining to oxidation of arsenite ions (eqn (2)) develop between 0.62 and 1.0 V as shown in Fig. 6B.

2 AsO_2_^−^ + 2H_2_O → AsO_4_^3−^ + 4H^+^ + 2e^−^

**Fig. 7:**
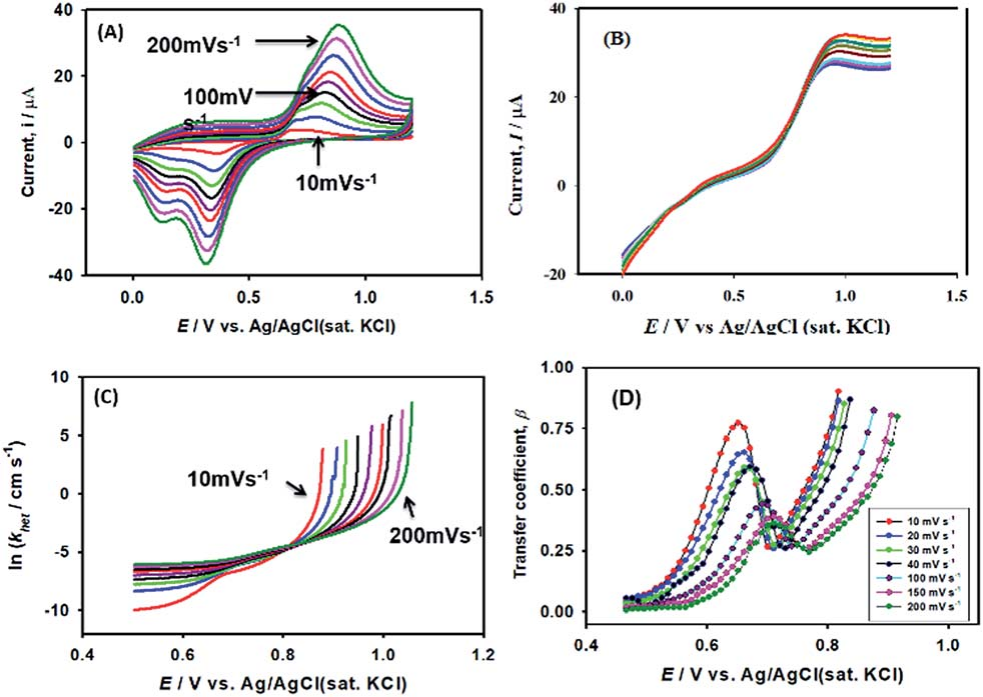
Influence of scan rates on heterogeneous electron transfer kinetics at Pt–Pd_sds_ electrode in 0.1 M H_2_SO_4_. (A) Cyclic voltammograms of 0.25 mM NaAsO_2_ at variable scan rates, (B) convoluted current (background current uncorrected), (C) potential dependency of heterogeneous rate constant and (D) potential dependency of transfer coefficient (*β*).

As expected, the most intense oxidation wave due to the reaction (eqn (2)) is observed in case of the Pt–Pd_sds_ electrode. This observation ensures that maximum active sites were generated over the Pt–Pd_sds_ surface to oxidize arsenite ions. It should be noted that at 0.72 and 0.80 V during the forward scan, two weak peaks are observed rather than a unique single peak. In order to assay these peaks, electro-kinetic investigations were performed next. For this purpose, CVs were recorded at variable scan rates (10 to 200 mV s^−1^) as shown in Fig. 7. It is observed from Fig. 7A that irrespective of the scan rate, a dual-hump like wave was retained during the positive scan. To explain such current–potential behaviour, convolution potential sweep voltammetry (CPSV) is often helpful. According to CPSV, the convolution current (*I*) is associated to the experimentally observed voltammetric current (*i*) as per the convolution integral (eqn (3)).^41–46^3
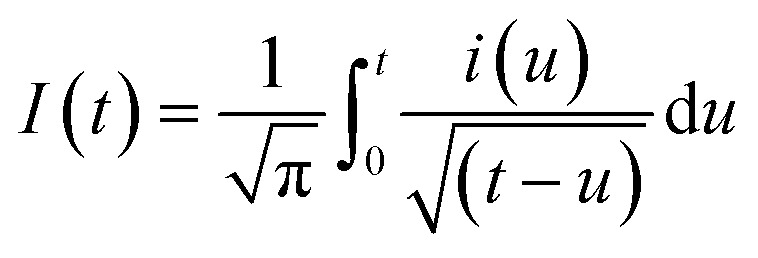


By approximating the above integral using Lawson–Maloy algorithm,^47^ the dependence of the convoluted current on applied potential can be deduced. In the present study, typical sigmoidal curves were obtained (shown in Fig. 7B) with a plateau at a large positive potential. The plateau level represents limiting convolution current (*I*_l_) as given by eqn (4).4
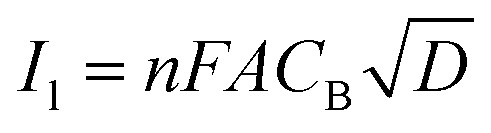


The heterogeneous rate constant (*k*_het_) for any irreversible electron transfer reaction can then be determined using the following equation (eqn (5)):^48,49^5
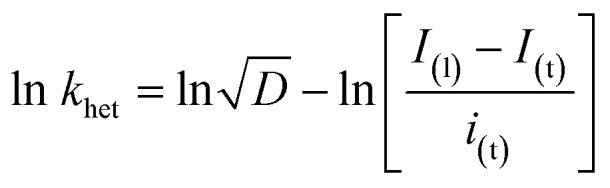
where, *I*_(l)_ is the convoluted limiting current, *i*_(t)_ is the cyclic voltammetric current and *I*_(t)_ is the convoluted current. As shown in Fig. 7C, the variation of ln(*k*_het_) with potential is non-linear, implying that the classical Butler–Volmer equation cannot be applied in such a case. As a consequence, the quadratic activation-driving force relation arising from the Marcus–Hush theory^50^ was employed, through which on derivatization of the ln(*k*_het_)–*E* plots, the apparent transfer coefficient *β*_app_ was obtained according to the linear regression of eqn (6) within a small potential range.6
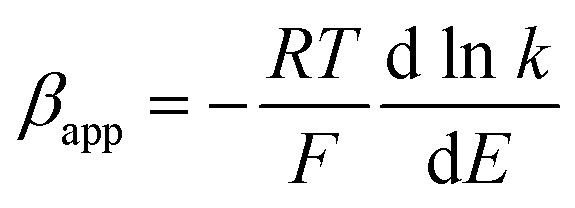


Using the heterogeneous rate constants extracted from eqn (5) at variable potentials, the values of *β* were calculated using eqn (6). Excitingly, as shown in Fig. 7D, the variation of *β* with applied potential is also non-linear consisting of two distinct linear segments denoting two different oxidations.

This behaviour is usually observed only when the oxidation reaction proceeds using a stepwise mechanism. Pertaining to CV obtained at 10 mV s^−1^ scan rate in the first segment before 0.65 V, As(iii) oxidized to metastable As(iv) species. In the second segment (above 0.70 V), intermediate As(iv) oxidized to As(v) species. The intermediate region between 0.65 and 0.70 V is critical, indicating that probably both oxidation reactions took place.

### Chronoamperometry

**Fig. 8:**
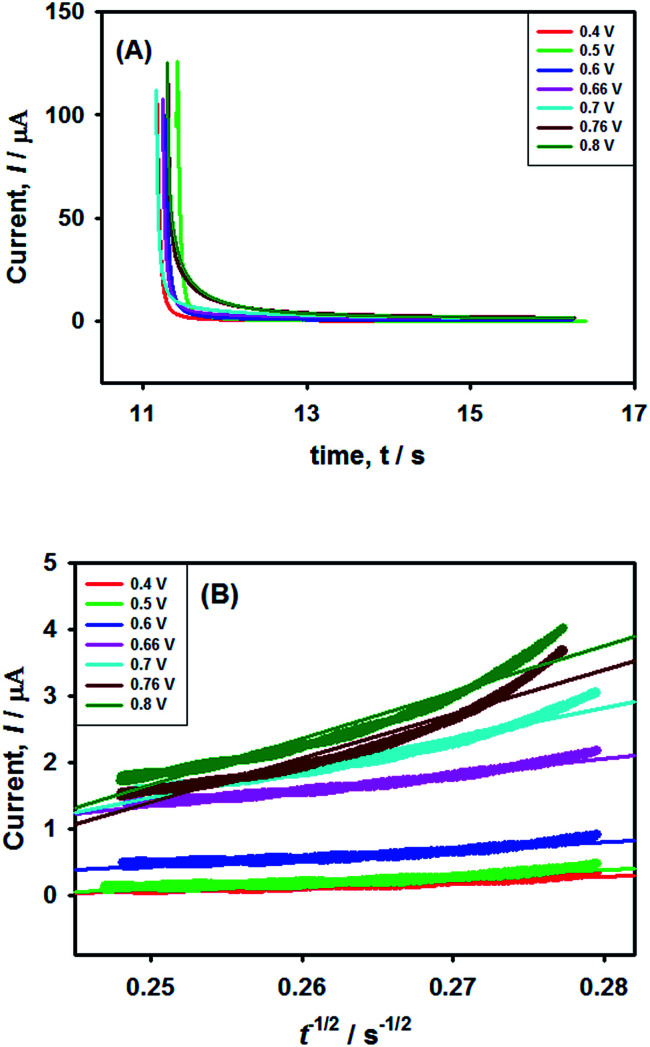
Chronoamperometric investigations of arsenite oxidation using Pt–Pd_sds_ electrode. (A) Chronoamperograms of 0.25 mM NaAsO_2_ in 0.1 M H_2_SO_4_ at different step potentials and (B) *t*^−1/2^*vs.* current graph according to the Cottrell equation.

In order to verify the results obtained by voltammetry, chronoamperometric (CA) investigations of 0.25 mM NaAsO_2_ were performed in 0.1 M H_2_SO_4_ applying different step potentials ranging between 0.4 and 0.8 V as shown in Fig. 8(A).

The changes in diffusional currents at different fixed potentials with respect to the reciprocal of the square root of time are illustrated in Fig. 8B. The currents are observed to increase with an increase in the step potential. Then, Cottrell equation [eqn (7)] was adapted to calculate the number of heterogeneous electron transfers:7
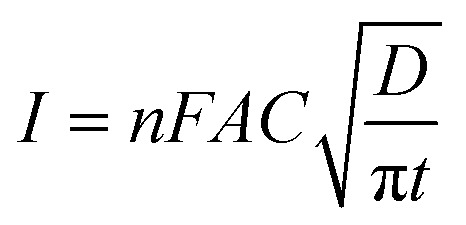
where, *I* is the faradic current (values taken from Fig. 8A), *n* is the heterogeneous electron transfer, *F* is the Faraday constant, *A* is the electrode active surface area (0.78 cm^2^), *C* is the bulk concentration of NaAsO_2_, *D* is the diffusion coefficient of AsO_2_^−^ (1.31 × 10^−5^ cm^2^ s^−1^), and *t* is the time. The CA data did not match well with eqn (7) at potentials below 0.60 V, but above this potential, the CA data provided important information concerning electron transfer kinetics. The slopes of the *I vs. t*^−1/2^ plots yielded the values of *n* to be 1.28 (∼1) for 0.70 V and 2.02 (∼2) for 0.80 V. This observation suggests that stepwise electron transfer was involved when arsenite ions were oxidized under the experimental conditions.

This result supports the results of the previously conducted CV and CPSV experiments, where we proposed that the electro-oxidation of As(iii) on the Pt–Pd_sds_ surface in a highly acidic medium is a two-step consecutive process. The As(iii) species releases one electron at just below 0.7 V under the experimental conditions to form a metastable As(iv) species, which instantly releases another electron and forms As(v). Thus, it can be concluded that As(iii) electro-oxidation undergoes *via* As(iv) to form As(v) at the Pt–Pd_sds_ electrode surface in 0.1 M H_2_SO_4_.

These observations are also supported by the study conducted by Catherino *et al.*, in which the authors analysed the *i*–*E* behaviour of As(iii) oxidation curves recorded by a Pt electrode in HClO_4_ and predicted a stepwise (As(iii) → As(iv) → As(v)) reaction path.^23^

### LOD evaluation

**Fig. 9:**
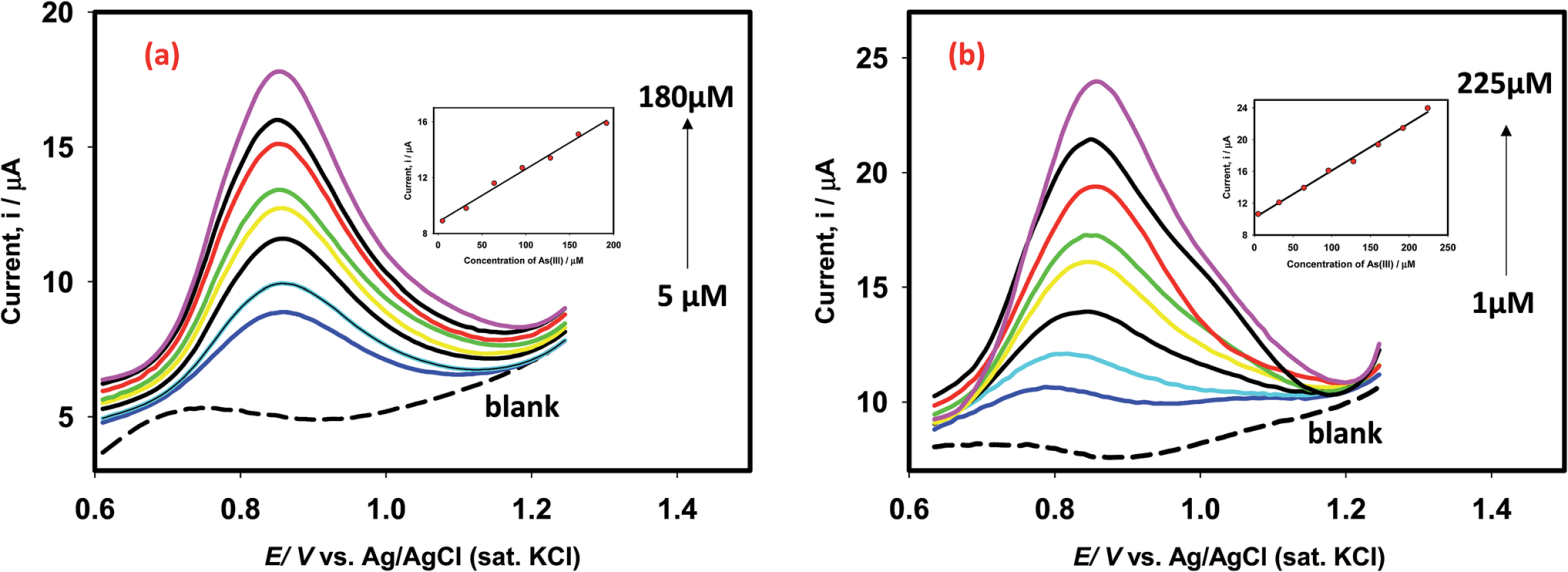
Square wave voltammograms of As(iii) detection in 0.1 M H_2_SO_4_ using Pt–Pd (a) and Pt–Pd_sds_ (b) electrodes. The square wave voltammograms were recorded under inert conditions using an optimized step potential of 8 mV, amplitude 20 mV and frequency 25 Hz. Inset shows the linear relationship of current *vs.* concentration.

In order to propose an efficient sensor for determination of As(iii) in drinking water, we obtained the square wave voltammograms (SWV) of arsenite ions using Pt–Pd and Pt–Pd_sds_ electrodes as shown in Fig. 9. For this purpose, the SWVs of variable arsenite concentrations were recorded between 1 μM and 225 μM (of As(iii) concentration) at room temperature. The Pt–Pd and Pt–Pd_sds_ electrodes exhibited a linear relationship (*I*_p_*vs.* conc.) within the reported concentration range (5 to 225 μM for Pt–Pd and 1 to 225 μM for Pt–Pd_sds_). The slopes of the plots were 4.0 × 10^−2^ AM^−1^(*r*^2^: 0.99) and 9.5 × 10^−2^ AM^−1^(*r*^2^: 0.99), for the Pt–Pd and Pt–Pd_sds_ electrodes, respectively. The larger slope value of the calibration curve exhibited by the Pt–Pd_sds_ electrode justifies its superior catalytic efficiency pertaining to arsenite oxidation reactions. The limit of detection (LOD) was obtained from the slope (SC) of the calibration curve and standard deviation (SD) of the blank solution (LOD = 3 × [SD/SC]). The evaluated LOD values were found to be 1.3 μg L^−1^ and 0.2 μg L^−1^ for Pt–Pd and Pt–Pd_sds_ electrodes, respectively. These results are much better than those obtained by the bulk Pt electrode.^27^ It should be noted that the WHO recommends the maximum permissible level of arsenic in drinking water to be 10 μg L^−1^. Thus, our experimental data suggest that the Pt–Pd_sds_ sensor might be helpful in determining arsenic for practical purposes compared to various types of Pt based electrodes as reported in Table 1.

**Table 1:** Comparison of the sensor performances for the determination of As(iii) using various Pt based electrodes^a^

Electrode systems	Technique/method	LDR/μM	LOD/μg L^−1^	Electrolyte	Ref.
Pt NPS	LSV	—	2.1	0.1 M H_2_SO_4_	27
Au–PtNPs/GCE	LSASV	0.375–225	0.28	0.5 M H_2_SO_4_	51
FePt NPs	SWASV	1–15	0.8	10 mM PBS (7.0)	52
PtNPs-C/GCE	SWASV	0–1000	—	0.5 M H_2_SO_4_	53
Pt–Pd	SWV	5–225	1.3	0.1 M H_2_SO_4_	This work
Pt–Pd_sds_	SWV	1–225	0.2	0.1 M H_2_SO_4_	This work

a Note: LSASV: linear sweep anodic stripping voltammetry; SWASV: square wave anodic striping voltammetry; LSV: linear sweep voltammetry; SWV: square wave voltammetry; LDR: linear dynamic range; LOD: Limit of Detection [LOD = 3 x (standard deviation of the blank/slope of the linear *I*_p_*vs.* conc. plot)].

### Determination of arsenite in real samples

To check the validity of the developed catalytic surface as a sensor in real system analysis, Pt–Pd_sds_ was employed to quantify arsenite from underground water by the standard addition method. Five underground water samples were collected from different deep-tube wells and solutions of arsenite ions (10.0 μg L^−1^) were prepared. The obtained results are summarized and presented in Table 2. A very good recovery of arsenite ions (99.6–102.3%) indicates the effectiveness and reliability of the proposed Pt–Pd_sds_ sensor.

**Table 2:** Quantification of arsenite in real samples (deep wells)

Samples	Added (μg L^−1^)	Obtained^a^ (μg L^−1^)	Recovery^b^ (%)	RSD^c^ (%)
Tube well-1	10	10.12	101.2	3.15
Tube well-2	10	9.99	99.90	3.12
Tube well-3	10	9.96	99.60	3.11
Tube well-4	10	10.23	102.30	3.17
Tube well-5	10	10.21	102.21	3.33

a Mean of the three repeated determinations (S/N = 3).

b (Concentration of arsenite determined/concentration of arsenite) × 100%.

c Relative standard deviation (RSD) value indicates precision among three repeated determinations.

Consequently, the present study suggests that performance of the Pd modified Pt electrode for arsenic detection is the best when the modification is performed in the presence of an SDS surfactant.

### Stability and interferences

**Fig. 10:**
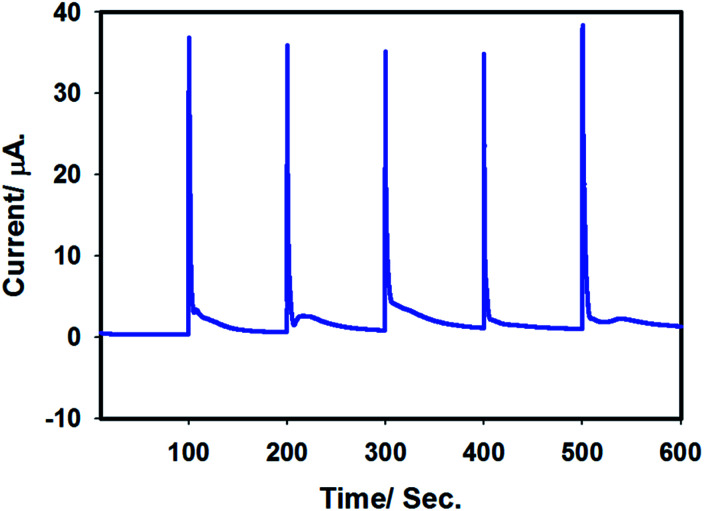
Batch injection chronoamperogram for stability test of Pt–Pd_sds_ in 0.1 M H_2_SO_4_. Applied potential: 0.9 V. Batch injection: 50 μL of 100 μM arsenite solution.

In order to check the stability of the proposed sensor, batch injection analysis was performed. For this, a homemade batch cell was used under choroamperometric mode. The experiment was carried out for 600 s by setting the working potential at 0.9 V in 0.1 M H_2_SO_4_. A constant current was assured before first injection of 50 μL of 100 μM arsenite solution. Periodically, arsenite solution was injected and the current intensity was increased, leading to sharp, reproducible peaks. A short time passed by the analyte at the surface of the electrode leads to a levelling of the signal for the presence of As(iii) species. The experiment was repeated several times and reproducible signals were observed as shown in Fig. 10. Thus, the batch chronoamperometry analysis showed that this sensor is stable and can be applied for longer duration for As(iii) detection.

Finally, interferences were checked. Arsenite primarily coexists in nature with Na^+^, K^+^, Fe^3+^, Cu^2+^, NO_3_^−^, SO_4_^2−^, Cl^−^*etc.* However, we did not notice any interference in the case of batch injection chronoamperometric experiments. This observation ensures that this sensor can be selectively used for As(iii) detection in water.

## Conclusion

Electro-oxidation of arsenite ions was investigated using cyclic voltammetry and CPSV techniques. A Pd modified Pt electrode surface was investigated using XPS analysis, which demonstrated that Pd exists both in metallic and oxidized states. The catalytic activity was significantly enhanced when Pd particles were deposited in the presence of a templating agent, *i.e.*, SDS. This may occur due to an increase in the porosity or catalytic active sites. The investigation of electron transfer kinetics revealed that arsenite oxidation reaction occurred using a stepwise reaction mechanism, forming a metastable As(iv) species in a certain potential range, which finally converted to As(v). The LOD of the As(iii) was determined to be 0.2 μg L^−1^ using a Pt–Pd_sds_ sensor. The result presented in this article, particularly enlargement of the active surface area of Pd nanoparticles may be beneficial to similar catalytic processes in future research.

## Conflicts of interest

There are no conflicts to declare.

## Supplementary Material

RA-008-C7RA12576C-s001
