# Supplementary material for: Electrochemical oxidation of As(iii) on Pd immobilized Pt surface: kinetics and sensing performance
Source: RSC Adv. 2018 Feb 20;8(15):8071–9. doi: 10.1039/c7ra12576c (PMC9078481; doi:10.1039/c7ra12576c)
Supplement: RA-008-C7RA12576C-s001 [file RA-008-C7RA12576C-s001.pdf]

Electronic Supplementary Material (ESI) for RSC Advances.
This journal is © The Royal Society of Chemistry 2018

Electronic Supplementary Material (ESI) for Analyst.
This journal is © The Royal Society of Chemistry 20XX

Electrochemical oxidation of As(III) on Pd immobilized Pt surface: Kinetics and sensing performance

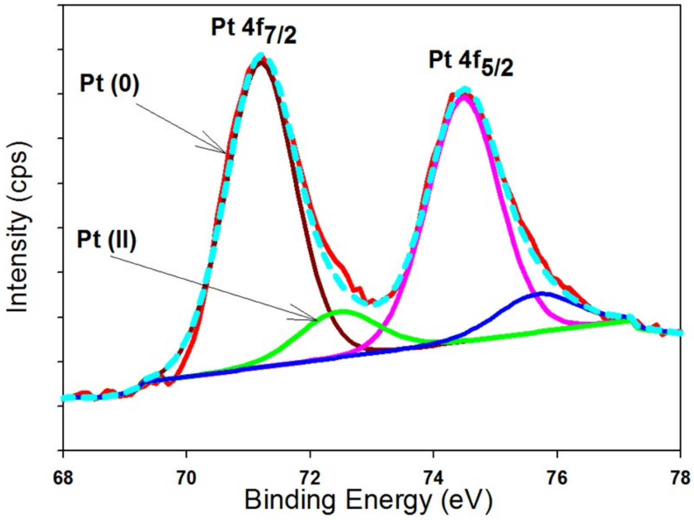

Fig.S1. XPS fine scan spectra of Pt particle (before Pd deposition)

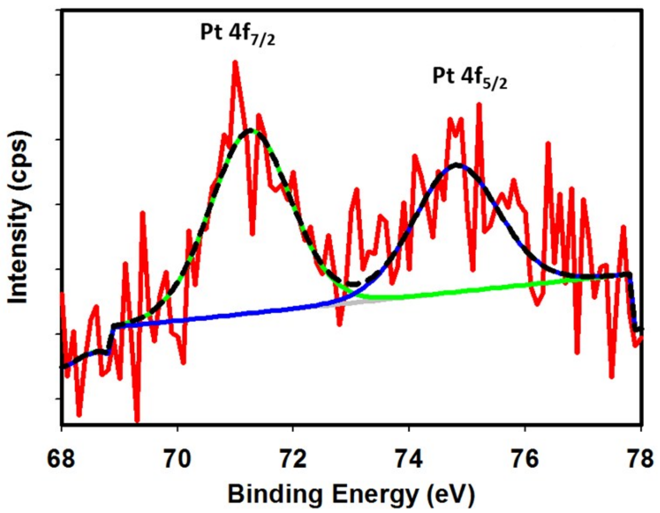

Fig.S2. XPS fine scan spectra of Pt particle (after Pd deposition as adlayers).

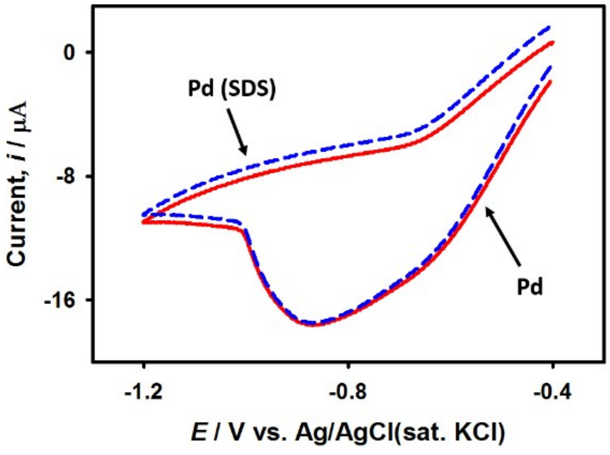

Fig.S3. Electrochemical deposition of Pd on Pt in presence (dotted line) and absence (solid line) of SDS molecules in 0.01 M $Pd(NO_3)_2$, scan rate at 100 mV s^{-1}.

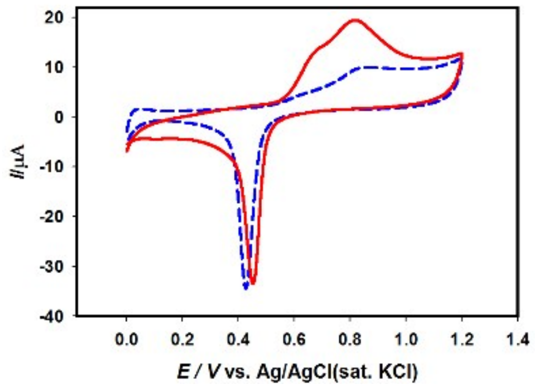

Fig. S4. CVs of Pd in 0.1 M H_2SO_4 (50.0 mVs^{-1}). (Dotted line blank and Solid line 0.25 mM $NaAsO_2$). Electrode diameter: 3.0 mm in diameter.
